# Hsa_circ_0038383-mediated competitive endogenous RNA network in recurrent implantation failure

**DOI:** 10.18632/aging.202590

**Published:** 2021-02-20

**Authors:** Huishan Zhao, Lili Chen, Yinghua Shan, Gang Chen, Yongli Chu, Huangguan Dai, Xuemei Liu, Hongchu Bao

**Affiliations:** 1 Reproductive Medicine Centre, The Affiliated Yantai Yuhuangding Hospital of Qingdao University, Yantai, China; 2 Department of Breast Surgery, The Affiliated Yantai Yuhuangding Hospital of Qingdao University, Yantai, China; 3 Department of Obstetrics and Gynecology, The Affiliated Yantai Yuhuangding Hospital of Qingdao University, Yantai, China

**Keywords:** recurrent implantation failure, circRNA, ceRNA, miRNA, endometrial receptivity

## Abstract

Background: Inadequate endometrial receptivity contributes to recurrent implantation failure (RIF) during IVF–embryo transfer. Though multiple circRNAs have been confirmed differentially expression in RIF, the potential function of novel circRNAs needed to be detected.

Results: The top ten DEcircRNAs were selected as initial candidates. A ceRNA network was conducted on the basis of circRNA–miRNA–mRNA potential interaction, consisting of 10 DEcircRNAs, 28 DEmiRNAs and 59 DEmRNAs. Three down-regulation circRNAs with high degree of connectivity were verified by RT-qPCR, and results suggested that only hsa_circ_0038383 was significantly downregulation in RIF compared with control group. Subsequently, three hub genes (HOXA3, HOXA9 and PBX1) were identified as hub genes. Ultimately, a subnetwork was determined based on one DEcircRNA (hsa_circ_0038383), two DEmiRNAs (has-miR-196b-5p and has-miR-424-5p), and three DEmRNAs (HOXA3, HOXA9 and PBX1). Following verification, hsa_circ_0038383/miR-196b-5p/HOXA9 axis may be a key pathway in affecting RIF.

Conclusion: In summary, a hsa_circ_0038383-mediated ceRNA network related to RIF was proposed. This network provided new insight into exploring potential biomarkers for diagnosis and clinical treatment of RIF.

Methods: We retrieved the expression profiles of RIF from GEO databases (circRNA, microRNA and mRNA) and constructed a competing endogenous RNAs (ceRNA) network based on predicted circRNA–miRNA and miRNA–mRNA pairs. The expression levels of three hub DEcircRNAs identified by cytoscape were validated by RT-qPCR.

## INTRODUCTION

Recurrent implantation failure (RIF), defined as a clinical symptom that implanted good quality embryos experience at least three times failure during *in vitro* fertilization-embryo transfer (IVF–ET), remains a challenging problem for assisted reproductive technology (ART) [1, 2]. However, a precise definition of RIF is still argumentative, leading to accurately recognize patients difficulty [3, 4]. The pathogenesis of RIF is various, relating to disturbed immune system, poor quality embryos and insufficient endometrial receptivity, among which poor endometrial receptivity is believed as the decisive cause of RIF [5, 6]. Currently, the receptivity of endometrium is evaluated by morphological features, but the invasive damage to childbearing age women during laparoscopy is inevitable. Therefore, seeking noninvasive diagnostic and prognostic biomarkers of RIF is urgently necessary.

Circular RNAs (circRNAs), as novel class of endogenous non-coding RNAs (ncRNAs), are covalently closed loop structures generated by a process named back-splicing with high stability, abundance and tissue specificity [7–9]. The loop structure of circRNAs makes it more suitable for biomarkers than their corresponding linear transcripts [10]. Recently, numerous of studies have suggested that circRNAs regulate gene expression by competitively binding as miRNA sponges, or interacted with RNA-binding proteins (RBPs) [11]. Growing evidences revealed that circRNAs were correlated with the diseases processes, containing immune disorder, tumorigenesis, metabolic disorders, degenerative disease, and disease occurrence and progression, etc [12–14]. Thus, circRNAs may become potential therapeutic target and biomarkers with diagnostic capabilities. Multiple studies have reported that the gene expression of endometrium is aberrant in RIF patients compared with healthy women, involving in coding RNA and non-coding genes [5, 15–17]. Yet, reports about focused on the differential expression of circRNAs in RIF are few. Only one study reported a set of differently expressed circRNAs (DEcircRNAs) in female with RIF for the first time [18]. Hence, discovering more RIF-related DEcircRNAs and exploiting their responding mechanism to understand pathogenesis and filter diagnostic biomarkers of RIF is necessary.

**Figure 1:**
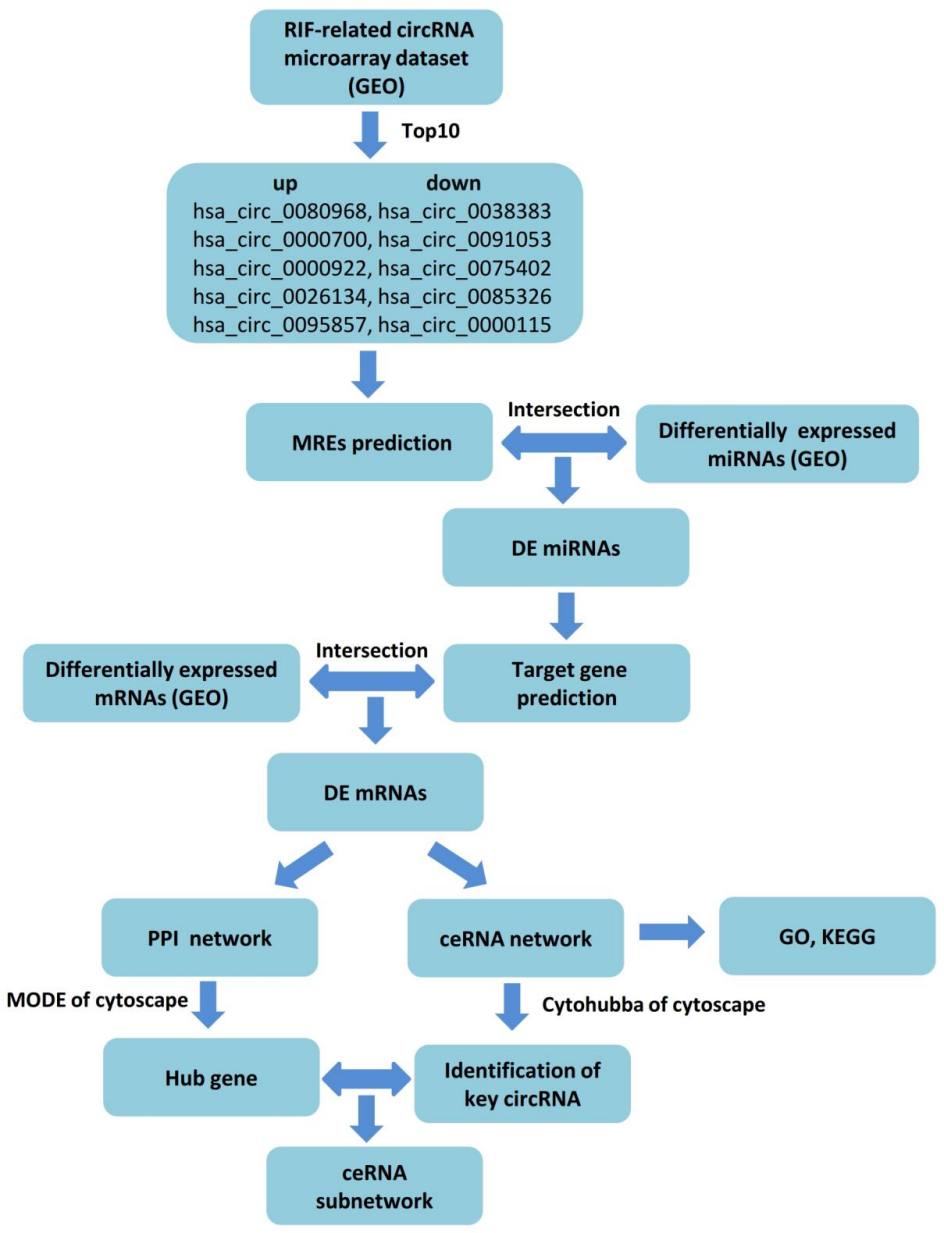
**Flow chart of the ceRNA network analysis.**

The competing endogenous RNA (ceRNA) hypothesis was described as ncRNAs regulating mRNA expression through competitively binding to shared miRNAs and forming a regulatory RNA network [19]. In recent years, it has been widely used in seeking candidate gene and exploring new mechanisms of circRNAs by constructing ceRNA regulated network based on bioinformatics analysis. Lu et al. found that hsa_circ_0011385 may play a key role in carcinogenesis-related pathways of bladder cancer as a ceRNA by identifying a circRNA–miRNA–mRNA regulatory network [20]. Another study disclosed that hsa_circ_0028883 could be regarded as a potentially reliable biomarker to diagnose active tuberculosis via comprehensive bioinformatics analysis and further verification with real-time quantitative polymerase chain reaction (RT-qPCR) [21]. In this study, we discussed the distinct circRNAs and their molecular mechanisms in RIF with the assistance of public databases. Firstly, we downloaded the expression profiles of circRNA (GSE147442), microRNA (GSE71332) and mRNA (GSE58144) of endometrium tissues of RIF patients and the control group from the Gene Expression Omnibus (GEO) database. Next, we selected the top 10 DEcircRNAs (5 upregulation and 5 downregulation) of GSE147442 as the initial candidates based on t-test. Then we established a circRNA–miRNA–mRNA regulation network depending on circRNA-miRNA interaction and miRNA–mRNA interaction by applying website tools and screening. Also, aim to expound the underlying mechanism of differently expressed mRNA (DEmRNAs) in ceRNA network, Gene Ontology (GO) analysis and Kyoto Encyclopedia of Gene and Genome (KEGG) pathway analysis were performed. Moreover, we chose three down-regulation circRNAs with a high degree of connectivity in ceRNA network as hub-circRNAs. While only hsa_circ_0038383 was identified with differential expression. Furthermore, hub genes of mRNA were screened out by a protein-protein interaction (PPI) network with Cytoscape MCODE (Molecular Complex Detection). Eventually, a circRNA-miRNA–mRNA subnetwork axis was successfully conducted. Thus, this research provided a forceful tool for searching potential biomarkers or therapeutic targets for RIF. A workflow summarizing our procedure is presented in Figure 1.

## RESULTS

### Identification of DEcircRNAs, differently expressed miRNAs (DEmiRNAs) and DEmRNAs in RIF endometrial tissues

**Figure 2:**
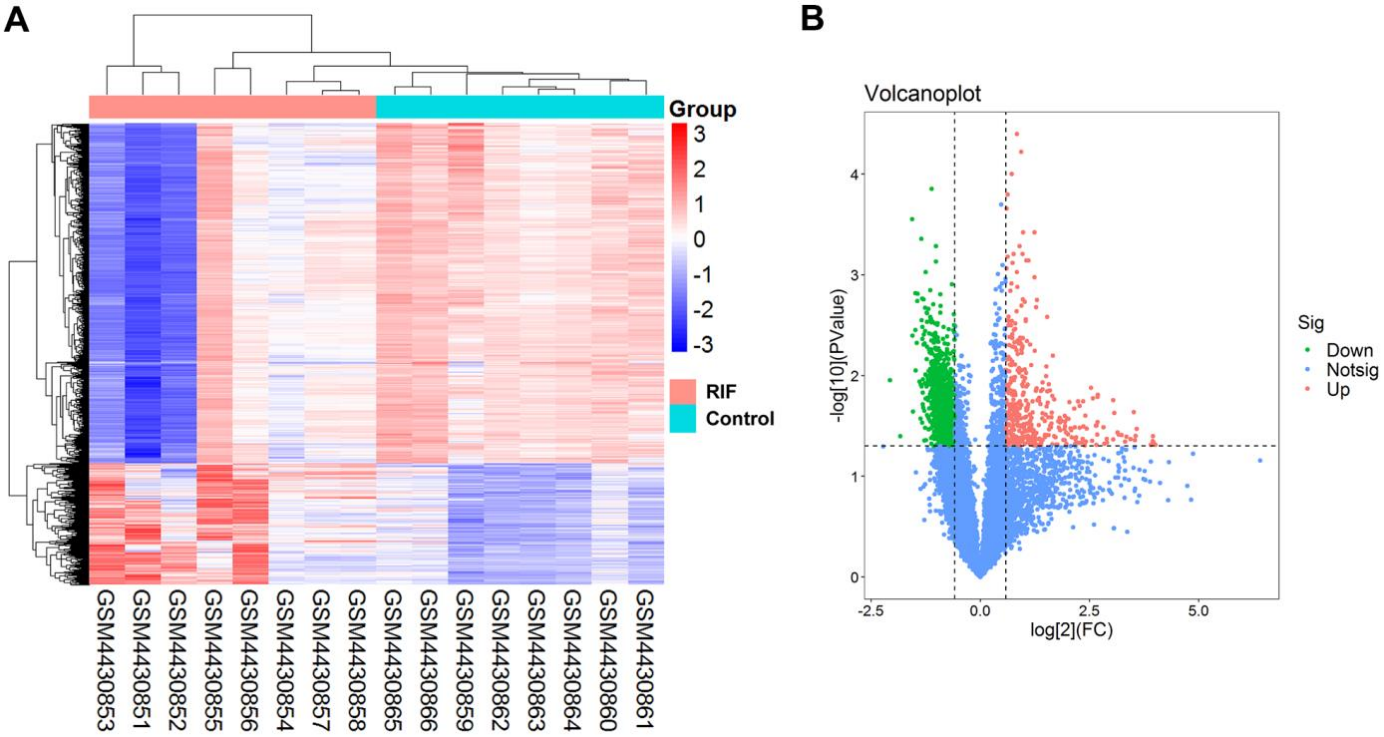
**Differentially expressed circRNAs in RIF patients compared the control group.** (**A**) Heatmap of the differentially expressed circRNAs in RIF based on GSE147442. (**B**) Volcano map for all circRNAs in GSE147442.

The basic information of three GEO datasets (GSE147442, GSE71332 and GSE58144) used in this study was shown in Table 1. 1468 DEcircRNAs (387 upregulation and 1081 downregulation) were filtered based on predetermined threshold in GSE147442. The DEcircRNAs were visualized by a volcano plot and a heat map (Figure 2A, 2B). The top ten DEcircRNAs in Table 2 were selected as initial candidates.

**Table 1:** Basic information of the three microarray datasets from GEO.

**Data source**	**Platform**	**Tissue**	**Sample size (RIF/control)**	**Author**	**Year**	**Region**	**RNA type**
GSE147442	GPL21825	Endometrium	8/8	Yan	2020	China	circRNA
GSE71332	GPL18402	Endometrium	7/5	Fan	2017	China	miRNA
GSE58144	GPL15789	Endometrium	43/42	van Hooff	2015	Netherlands	mRNA

**Table 2:** Basic characteristics of the top ten DEcircRNAs.

**CircRNA ID**	**FC**	**P value**	**Gene symbol**	**Regulation**
hsa_circ_0080968	3.17	0.0064	ADAM22	Up
hsa_circ_0000700	2.89	0.0026	CHD9	Up
hsa_circ_0000922	2.85	0.0081	ZNF536	Up
hsa_circ_0026134	2.46	0.0018	TUBA1C	Up
hsa_circ_0095857	2.42	0.0021	PRDM11	Up
hsa_circ_0000115	0.34	0.0003	CSDE1	Down
hsa_circ_0085326	0.34	0.0040	EIF3E	Down
hsa_circ_0075402	0.36	0.0015	GNB2L1	Down
hsa_circ_0091053	0.36	0.0035	RPS4X	Down
hsa_circ_0038383	0.36	0.0089	THUMPD1	Down

**Figure 3:**
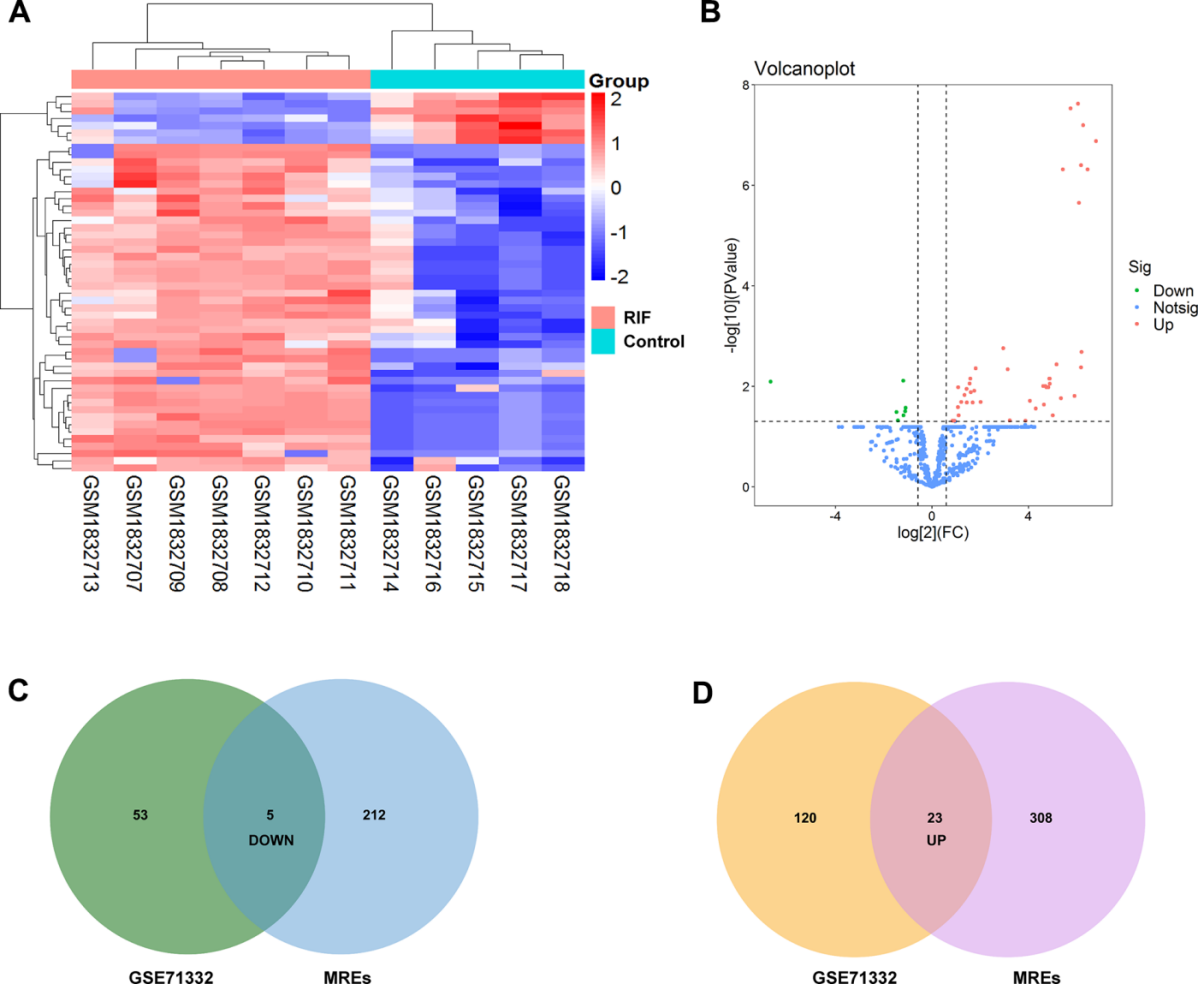
**Identification of differentially expressed miRNAs.** (**A**) Heatmap of the differentially expressed miRNAs from the GEO microarray GSE71332. (**B**) Volcano map for all miRNAs in GSE71332. (**C**, **D**) Overlapping between circRNA-related target miRNAs predicted by online tool and DEmiRNAs in GSE71332.

After batch effect normalization and further analyses using a limma (versions 3.30.0) R package, we screened out 143 upregulation miRNAs and 58 downregulation miRNAs with p<0.05 from GSE71332 (Figure 3A, 3B). According to the above top ten DEcircRNAs, circRNA–miRNA pair prediction was conducted using online tool CircInteractome and ENCORI database. 217 target miRNAs based on 5 upregulation circRNAs and 331 target miRNAs based on 5 downregulation circRNAs were obtained. Subsequently, the predicted MREs of circRNAs and DEmiRNAs were intersected to acquire 5 down- and 23 upregulation miRNAs using Venn software online (Figure 3C, 3D).

**Figure 4:**
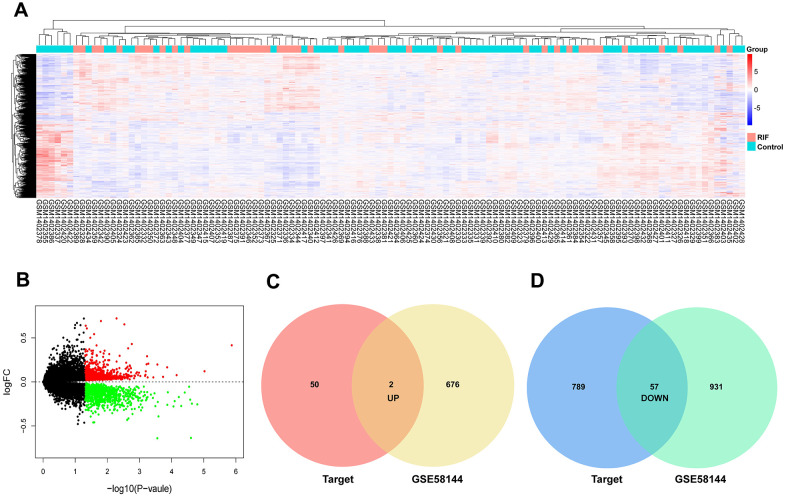
**Identification of differentially expressed mRNAs.** (**A**) Heatmap of the differentially expressed mRNAs in GSE58144. (**B**) Volcano map for all mRNAs in GSE58144. (**C**, **D**) Venn diagram analysis of DEmRNA-predicted targets and differentially expressed mRNAs in GEO.

Using the same data processing method, 678 upregulation and 988 upregulation mRNAs in GSE58144 were selected (Figure 4A, 4B). On basis of similar methods, 59 candidate mRNAs were chosen, of which 2 were upregulated and 57 were downregulated (Figure 4C, 4D).

### Construction of the ceRNA network

**Figure 5:**
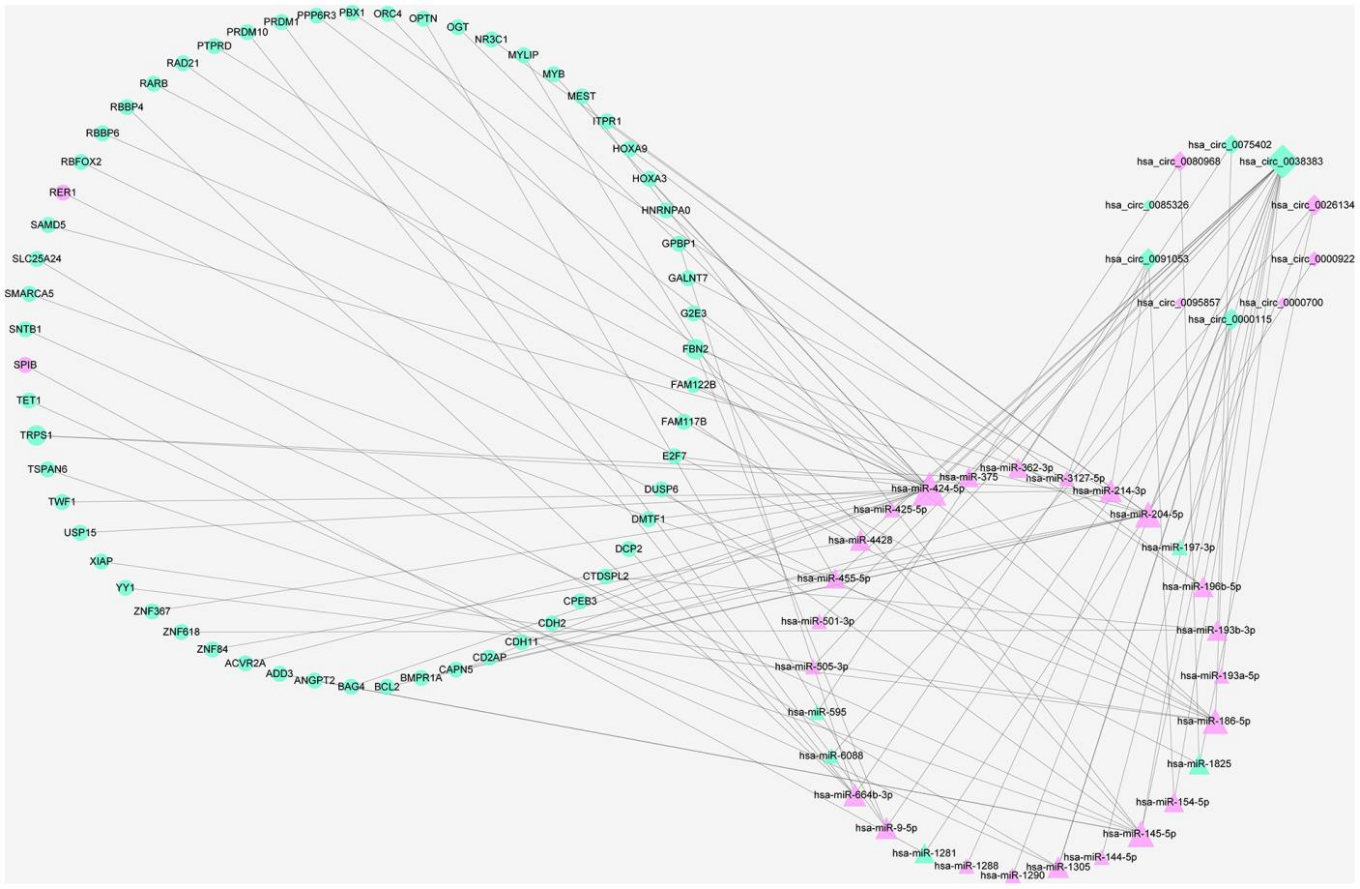
**The ceRNA network of circRNA–miRNA–mRNA in RIF.** Diamonds represent circRNAs, triangles indicate miRNAs, and rounds indicate mRNAs. Node size represent the degrees of node in the ceRNA network. The node of red and green color express upregulation and downregulation, respectively.

To better comprehend the role of circRNAs in miRNAs mediated mRNAs, a circRNA–miRNA–mRNA regulatory network was generated employing a combination of circRNA–miRNA pairs and miRNA–mRNA pairs after multiple screening steps. The ceRNA contained 33 circRNA–miRNA pairs and 63 miRNA–mRNA pairs, including 10 DEcircRNAs, 28 DEmiRNAs and 59 DEmRNAs. The subnetwork was presented using cytoscape software (Figure 5).

### GO and KEGG enrichment analyses

**Figure 6:**
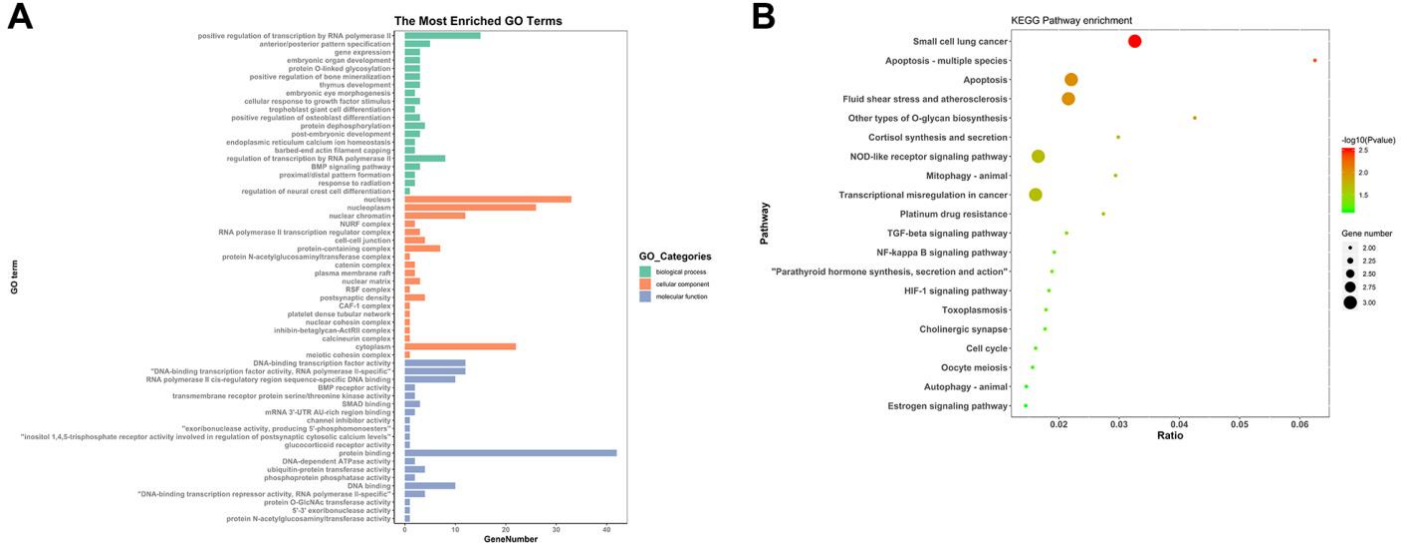
**GO and KEGG analyses of 59 mRNAs.** (**A**) GO analysis. (**B**) KEGG pathway analysis.

To find out the biological functions and pathway of DEmRNAs in ceRNA network, GO annotation and KEGG pathway analysis was performed. GO enrichment analysis was constituted with biological processes, molecular functions and cellular components. In biological process terms, the DEmRNAs were primarily involved in ‘regulation of transcription’ and ‘gene expression’. For cellular component terms, the DEmRNAs were mainly enrichment in ‘nucleus’, ‘nucleoplasm’, ‘transcription regulator complex’ and ‘cell-cell junction’. While ‘transcription factor activity’, ‘BMP receptor activity’, ‘transmembrane receptor protein serine/threonine kinase activity’ and ‘SMAD binding’ were mainly related to molecular function (Figure 6A). As shown in Figure 6B, KEGG pathway analysis indicated that the 59 aberrant DEmRNAs in RIF were significantly enriched in ‘apoptosis’, ‘fluid shear stress and atherosclerosis’, ‘NOD-like receptor signaling pathway’, ‘Mitophagy’, ‘TGF-beta signaling pathway’ and ‘NF-kappa B signaling pathway’, etc. (Figure 6B).

### Identification and verification of hub DEcircRNAs

**Figure 7:**
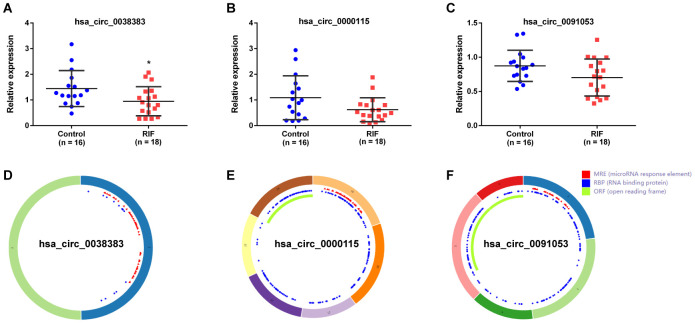
**Expression verification and structure of circRNAs.** The expression of hsa_circ_0038383 (**A**), hsa_circ_0000115 (**B**) and hsa_circ_0091053 (**C**) in RIF compared with the controls. Structural patterns of hsa_circ_0011385 these three DEcircRNAs by circRNADb.

CircRNAs act as hub node in biological networks. Three down-regulation circRNAs (hsa_circ_0038383, hsa_circ_0000115 and hsa_circ_0091053) with high degree of connectivity calculated by the cytoHubba plugin of cytoscape were filtered as hub-circRNAs. Their basic characteristics were expressed in Table 3. To further assess the expression of these three selected hub DEcircRNAs, 18 RIF patients and 16 healthy women were included as a validation cohort. The detailed characteristics of these patients were summarized in Table 4. Full-length sequences were got from CircInteractome website, and sequences of specific primers for each circRNA were designed by Primer-BLAST in PubMed. The primers information was shown in Table 5. It was noteworthy that only hsa_circ_0038383 was significantly downregulated in endometrial tissues of RIF patients compared with control groups (Figure 7A). Hsa_circ_0000115 and hsa_circ_0091053 showed no significant difference (Figure 7B, 7C). The basic structural patterns of the three circRNAs were displayed in Figure 7D–7F. Consequently, hsa_circ_0038383 was selected as the circRNA target for future research.

**Table 3:** Basic characteristics of the three selected differently expressed circRNAs.

**CircRNA ID**	**Position**	**Strand**	**Genomic length**	**Best transcript**	**Gene symbol**	**Regulation**
hsa_circ_0038383	chr16:20744988-20749278	-	4290	NM_017736	THUMPD1	Down
hsa_circ_0000115	chr1:115276352-115280693	-	4341	NM_001242892	CSDE1	Down
hsa_circ_0091053	chrX:71492452-71496084	-	3632	NM_001007	RPS4X	Down

**Table 4:** Clinical characteristics of women recruited in the present study.

**Variables**	**Control groups (n=16)**	**RIF groups (n=18)**	***p* value**
Age (years)	30.19±2.79	30.89±3.43	0.52^a^
BMI (kg/m^2^)	21.14±1.57	22.01±2.21	0.20^a^
FSH (mIU/ml)	7.53±2.02	7.25±1.56	0.66^a^
LH (mIU/ml)	4.68±2.08	4.01±2.03	0.37^a^
Estradiol (pg/ml)	38.60±10.76	34.87±11.13	0.33^a^
Infertility duration (years)	4.16±1.79	3.64±2.26	0.43^a^
Number of embryo transfer cycles	1(14/16, 87.5%) 2(2/16, 12.5%)	3(9/18, 50.0%) 4(4/18, 22.2%) 5(3/18, 16.7%) 6(2/18, 11.1%)	<0.001^b^
Number of embryos per transfer	1.50±0.63	1.61±0.61	0.61^a^

^a^Student t-test; ^b^Fisher’s exact test.

Data presented as mean ± SD. *P* < 0.05was considered statistically significant.

**Table 5:** Primer sequences of circRNAs for RT-qPCR.

**circRNAs**	**Forward primer (5’-3’)**	**Reverse primer (5’-3’)**
hsa_circ_0038383	TACGGCCAGAAATCGAGCTT	ATGTGCCTGAGATGGGTAACA
hsa_circ_0000115	TGCCTCAAGGAACAGTCATT	AATGGGTTTCCCAGTCCGTC
hsa_circ_0091053	GAGCAGTGGGTGAAATGGGT	ATGGACGAGGAGCAAACACA
hsa-miR-196b-5p	CGCGCTAGGTAGTTTCCTGTTGTTGGG	Tailing Reaction, Tm (65° C)
hsa-miR-424-5p	CGCGCGCAGCAGCAATTCATGTTTTG	Tailing Reaction, Tm (65° C)
U6	CGCAGAGAAGATTAGCATGGCCCCTG	Tailing Reaction, Tm (65° C)
HOXA9	TAAACCTGAACCGCTGTCGG	CCAGTTGGCTGCTGGGTTAT
PBX1	GCTGATGCATTCCCATGCTG	TGGGCTCCTCGGATACTCAA
HOXA3	GCGACCTACTACGACAGCTC	CTCACTCAGTTCGTGTGCCT
β-actin	CTGGACTTCGAGCAAGAGATG	GAGTTGAAGGTAGTTTCGTGGA

### PPI network construction and hub mRNA validation

**Figure 8:**
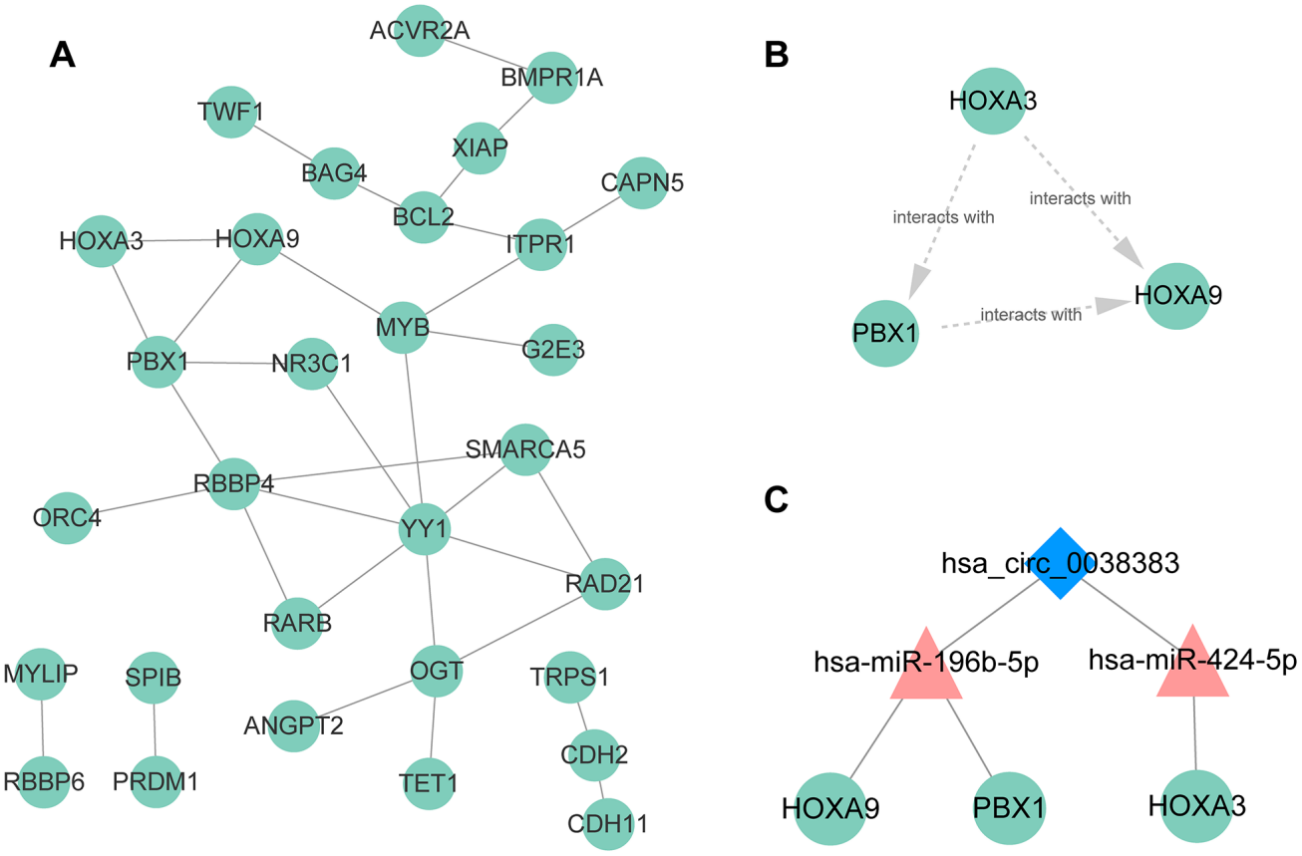
**PPI network, hub genes, and circRNA–miRNA–mRNA subnetwork for hsa_circ_0038383.** (**A**) PPI network of 59 DEmRNA. (**B**) Three hub genes extracted from the PPI network based on the MCODE algorithm. (**C**) The circRNA-miRNA-mRNA axes of hsa_circ_0038383.

**Figure 9:**
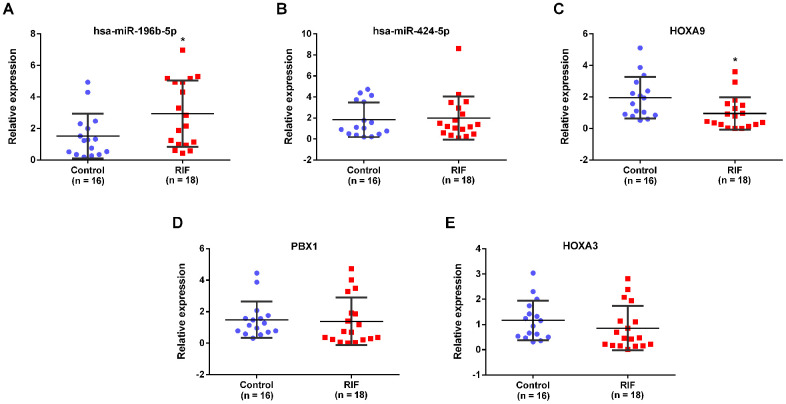
**Expression verification of miRNAs and mRNAs in the subnetwork.** The expression of has-miR-196b-5p (**A**), has-miR-424-5p (**B**), HOXA9 (**C**), PBX1 (**D**) and HOXA3 (**E**) in endometrial tissues of RIF and the control group.

The PPI network was established based on protein interaction provided by STRING database. 30 nodes and 134 edges were included in this PPI network (Figure 8A). Then the most significant cluster in this PPI network was uncovered applying cytoscape MCODE module. The results indicated that only one cluster as key genes, including PBX1, HOXA9 and HOXA3 (Figure 8B). Based on the above results, we constructed a circRNA–miRNA–mRNA subnetwork, consisting of one DEcircRNA (hsa_circ_0038383), two DEmiRNAs (has-miR-196b-5p and has-miR-424-5p), and three DEmRNAs (HOXA3, HOXA9 and PBX1), which provided a novel insight in the pathogenesis and treatment of RIF (Figure 8C). Aimed to confirm whether the expression level of miRNAs and mRNAs in the particular axis is in accordance with prediction, we detected their expression in endometrial tissues of RIF patients compared with the healthy women. The results showed that only has-miR-196b-5p and HOXA9 was consistent the predicted results (Figure 9A–9E), which pointed that the hsa_circ_0038383/miR-196b-5p/HOXA9 axis may be a key pathway leading to RIF.

## DISCUSSION

RIF is a major cause of female infertility and a challenging problem in assisted reproduction field. In addition, the pregnancy rate after the therapy of IVF-ET still remains approximate 25% [22]. Good endometrium receptivity during the window of implantation is necessary for successful embryo transfer. Aberrant gene expression is a key factor in causing insufficient endometrium receptivity, involving in coding and non-coding gene. Wang et al. [23] found several mRNAs which may affect embryo implantation, and identified one possible target in endometrial tissues may be used for treating RIF based on GSE58144. Another study demonstrated that 15 lncRNAs abnormally expressed in endometrial tissues could be regarded as potential biomarkers for RIF through performing gene sequencing and further RT-qPCR verification [24]. Additionally, six key lncRNAs were screened out and their ceRNA subnetworks were conducted by comprehensive bioinformatics analysis, which signified lncRNAs could be developed into predictive biomarkers for RIF [25]. The high stability circRNAs with cyclic structures, exhibit specific expression in tissue or developmental stage and make this type of ncRNAs more valuable as new biomarkers for diverse diseases. Recently, a study showed that several circRNAs were existed with different expression between RIF patients and normal controls, which may furnish diagnosis molecular and therapeutic target for RIF [18].

Nowadays, more and more attention was attracted in investigating the underlying functions and mechanisms of circRNAs through establishing ceRNA network. For instance, in the research of lung adenocarcinoma, Liang et al. [26] appraised significant circRNAs and meaningful circRNA–miRNA–mRNA networks based on the guidance of bioinformatics. Also, hsa_circ_0001955/hsa_circ_0000977-mediated ceRNA sub-network was confirmed to uncovering potential target of colorectal cancer by whole-transcriptome analysis [27]. Furthermore, circUBAP2-mediated circRNA–miRNA–mRNA network modulated the development and progression of pancreatic adenocarcinoma via regulating the infiltration and function of immune cells [28]. In addition, a circRNA-miRNA-hub gene axis was constructed to discover potential therapeutic targets for spinal cord injury by way of microarray data mining and comprehensive bioinformatics analyses [29].

To further understand the potential molecular mechanism and functions of circRNAs on RIF, we constructed a circRNA–miRNA–mRNA regulatory network by differential expression analysis, intersection analysis and correlation analysis. The DEmRNAs involving in biological progress and pathway were performed by GO and KEGG analysis. Although there were few reports on the GO terms directly related to RIF, the GO terms related to endometrial receptivity have been gradually discovered. The GO analysis showed BMP signaling pathway and SMAD binding are two important terms. For example, BMP mediated endometrial receptivity and decidualization [30]. Additionally, smad dependent TGF-β signaling acted a major role in the process of embryo implantation. KEGG pathway analysis indicated that Apoptosis, TGF-beta signaling pathway and signaling pathway. Yu et al. found that the apoptosis of HIF-1α affected embryo implantation during implantation window of women with RIF [31]. Another study indicated that TGF-β1 was down-regulated in patients with RIF and played an important role in endometrial receptivity and embryo implantation [32]. Moreover, Ersahin A et al. found that the expression of endometrial NF-κB expression was disturbed in women with RIF [33]. Therefore, the GO terms or KEGG pathways provided direction for the future mechanism research. Subsequently a circRNA–miRNA–mRNA subnetwork was conducted, of which hsa_circ_0038383 may work as a ceRNA to capture hsa-miR-196b-5p in regulating the expression of HOXA9 and PBX1. Also, hsa_circ_0038383 may positively modulate expression of HOXA3 by acting as hsa-miR-424-5p sponge. These results revealed that the circRNA–miRNA–mRNA network of hub-circRNA not only be a novel candidate biomarker, but also provided evidence for regulation mechanism of circRNA.

Hsa_circ_0038383/miR-196b-5p/HOXA9 and hsa_circ_0038383/miR-196b-5p/PBX1 were two essential axes from the subnetwork. The verification results revealed that only the hsa_circ_0038383/miR-196b-5p/HOXA9 axis may be a key pathway leading to RIF. HOXA9 (Homeobox A9), a homeodomain transcription factor, was a regulator of embryonic development similar to HOX members [34]. Recently, its role in endometrium was gradually reported. The expression of HOXA9 was increased during mid-secretory phase of menstrual cycle and silence of Hoxa9 in the murine uterus reduced average implantation rates [35]. This result prompted that HOXA9 low expression could result in decreasing endometrial receptivity. In our study, the expression of HOXA9 should be downregulated in RIF with low endometrial receptivity, which was positively with hsa_circ_0038383. These consequences suggested that our research was consistent with other study. HOXA9 as the target of hsa-miR-196b-5p, the targeting relationship between them had been confirmed by multiple studies. One study reported that miR-196b directly targeted HOXA9 adjusting aggressiveness through NF-κB activity in non-small cell lung cancer cells [36]. Another study showed that in mixed lineage leukaemia (MLL)-rearranged leukaemia, miR-196b could directly target left HOXA9/MEIS1 oncogenes and FAS tumor suppressor [37]. However, the relationship between miR-196b and PBX1 was still unknown. Therefore, we speculated that hsa_circ_0038383 influenced uterine receptivity and embryo implantation by miR-196b/HOXA9 axis, and we would test this axis in the future.

Hsa_circ_0038383/hsa-miR-424-5p/HOXA3 was also included in the subnetwork. The expression of miRNAs and target genes between menstrual endometria and early pregnancy were compared, of which miR-424-5p were significantly downregulated during early pregnancy deciduas [38]. But related research about HOXA3 in endometrial receptivity and the target relationship between HOXA3 and miR-424-5p were still unknown. Moreover, the correlation of genes expression in hsa_circ_0038383/hsa-miR-424-5p/HOXA3 axis was not confirmed in our research. Whether this axis played roles in endometrium acceptability needed more experiments.

In this study, a novel circRNA–miRNA–mRNA network related with RIF was proposed and hsa_circ_0038383 imbalance was verified in RIF. This network would contribute to explore the initiation and progression of RIF and further develop potential treatment strategies for this disease. However, in view of the results are mainly based on computational biology and RT-qPCR, further biological and molecular experiments are indispensable to verify our hypothesis.

## CONCLUSIONS

In summary, we constructed a potential hsa_circ_0038383-mediated ceRNA subnetwork which provided new insight into exploring molecular mechanism and offering a candidate biomarker for RIF. Especially, hsa_circ_0038383/miR-196b-5p/HOXA9 axis may be a key pathway in affecting uterine receptivity and embryo implantation, and further in-depth molecular biology experiments on this axis are necessary to verify the circRNA role in RIF. This study contributed to seeking new ideas for diagnostic biomarkers and therapeutic targets for patients with RIF.

## MATERIALS AND METHODS

### Microarray data information

The raw data of circRNA, microRNA and mRNA were obtained from the GEO http://www.ncbi.nlm.nih.gov/geo/) dataset, which is an international public repository and recording platform employed for searching any appropriate datasets. GSE147442 is a circRNAs database of RIF and based on the GPL21825 platform, containing 8 RIF endometrial and 8 normal endometrium specimens. GSE71332 is a miRNAs database of RIF and based on the GPL18402 platform, consisting of 7 RIF endometrial and 5 normal endometrium specimens. GSE58144 is a mRNAs database of RIF and based on the GPL15789 platform, involving in 43 RIF endometrial and 42 normal endometrium samples. The fundamental information of this gene chip was shown in Table 1. DEcircRNAs were identified based on the fold-change and Student’s t testing (p<0.05, FC>1.5). While data in GSE71332 and GSE58144 were normalized and processed using bioconductor limma (versions 3.30.0) R package. DEmiRNA and DEmRNA were acknowledged with p value <0.05. The basic information of these three GEO datasets used in this study was shown in Table 1.

### Prediction of circRNA–miRNA pairs

The circBase website (http://www.circbase.org/) was utilized to observing the basic information of circRNAs [39]. The target miRNAs and miRNA response elements (MREs) presented in circRNAs were predicted by The Circular RNA Interactome (CircInteractome, https://circinteractome.nia.nih.gov/) [40] and the Encyclopedia of RNA Interactomes (ENCORI database, http://starbase.sysu.edu.cn/index.php) [41]. The overlapping miRNAs were further screened as potential target miRNAs to the DEcircRNAs based on the corresponding to target miRNAs to DEcircRNAs and DEmiRNAs of GSE71332.

### Prediction of miRNA–mRNA pairs

The miRNA–mRNA interaction were predicted with three online websites respectively, including TargetScan (http://www.targetscan.org/) [42], miRDB (http://www.mirdb.org/) [43] and miRTarBase [44]. Only target mRNAs recognized consistently by these three databases were selected as candidate targets and further were used to intersect with differentially expressed mRNAs of GSE58144 to screen out potential target mRNAs for the miRNAs.

### Construction of the circRNA–miRNA–mRNA network

To better uncover the correspondence among circRNAs, miRNAs and mRNAs, we constructed a circRNA–miRNA–mRNA regulatory network utilizing a combination of circRNA-miRNA pairs and miRNA–mRNA pairs. And the ceRNA network was visualized using cytoscape (http://cytoscape.org/; version 3.7.1) software [45].

### GO term and KEGG pathway enrichment analysis

The definitions of GO and KEGG analysis was described in our previous study [46]. To evaluate the main function pathways of RIF, DEmRNAs in ceRNA network were performed through GO annotation and KEGG pathway analyses. The results were depicted using clusterProfiler (versions 3.18.0) package in R (R-3.6.0).

### Construction of PPI network and identification of hub genes

The PPI network was established for indicating the interaction among the determined DEmRNAs more intuitively, according to the Search Tool for the Tetrieval of Interacting Genes (STRING) database (http://string-db.org/, version 11.0) [47]. And then the result was shown using cytoscape software. Subsequently, the hub mRNAs were appraised by cytoscape MCODE module, which is a plug-in used to find closely connected nodes in a complex network based on topology.

### Endometrial tissues

34 women (aged 24-40) treated at Reproductive Medicine Center of Yantai Yuhuangding Hospital were enrolled in our study. All of them signed informed consent forms approved by the Institutional Review Board of Yantai Yuhuangding Hospital (reference number 2019-121). The endometrial tissue samples in mid-secretory phase were obtained from RIF patients (n = 18) and control groups (n = 16), respectively. The women still did not get pregnancy after at least three IVF–ET failure cycles were assigned as RIF patients. The control women who experienced IVF–ET cycle due to tubal obstruction without hydrosalpinx achieved a clinical pregnancy after their first or second embryo transfer. Endometrium-related diseases, hydrosalpinx, polycystic ovarian syndrome (PCOS), etc. were excluded. All participants hold normal hormone level, normal endometrial thickness and morphology and a regular menstrual cycle (28-31 days).

### RNA extraction and RT-qPCR

Total RNA was isolated from the tissue samples using Trizol reagent following manufacturer’s instructions (Sparkjade, Qingdao, China). Then 1 μg RNA of each sample was reverse-transcribed to obtain cDNA using SPARKscript II RT Plus Kit (With gDNA Eraser) (Sparkjade, Qingdao, China). The expression of circRNAs and mRNAs in these individual samples was performed by qRT-PCR reaction using SYBR Green qPCR Mix kit (With ROX) (Sparkjade, Qingdao, China) following: 94° C for 2 min, followed by 40 cycles of 95° C for 10 s and 60° C for 30 s. The extraction and amplification of miRNAs was performed according to the manufacturer’s instructions (SPARKeasy animal tissue/Cell microRNA kit, miRNA first strand synthesis kit and miRNA SYBR Green qPCR Mix kit, Sparkjade, Qingdao, China). All RT-qPCR were repeated three times. The relative expression of all genes was calculated using the 2^-ΔΔCT^ method.

### Statistical analysis

The continue variables were expressed with means ± standard deviation. The statistical analysis of circRNAs was performed using GraphPad Prism (GraphPad Software, Inc., La Jolla, CA). The significance between groups was tested by Student’s t test or Fisher’s exact test. When p<0.05 were considered as statistically significant.
